# Detection of Marine Litter on Arctic Beaches Based on Aerial Photography Using the YOLOv8s CNN

**DOI:** 10.3390/s26196155

**Published:** 2026-09-28

**Authors:** Elizaveta Serdiukova, Aleksandr Danilov, Alexandra Ershova

**Affiliations:** 1 Department of Geoecology, Saint Petersburg Mining University, Saint Petersburg 199106, Russia; danilov_as@pers.spmi.ru; 2 PlasticLab, Russian State Hydrometeorological University, Saint Petersburg 192007, Russia; plasticlab.2019@gmail.com

**Keywords:** unmanned aerial system (UAS), deep learning, marine litter, plastic, environmental monitoring, convolutional neural network

## Abstract

**Highlights:**

**What are the main findings?**

- The YOLOv8s-OBB convolutional neural network, trained on a unique dataset of 1039 aerial images from the Novaya Zemlya archipelago, achieved a mean Average Precision (mAP50) of 0.709 and precision of 0.88 for detecting five classes of marine litter (“Metal”, “Plastic”, “Fishing gear”, “Wood”, and “Other”).
- The best detection performance was obtained for “Metal” (mAP50 = 0.811) and “Plastic” (mAP50 = 0.789), while “Wood” (mAP50 = 0.551) and “Other” (mAP50 = 0.556) proved to be the most challenging classes due to visual similarity to natural background objects and class heterogeneity.

**What are the implications of the main findings?**

- The integration of UAS-based aerial photography with deep learning provides a reliable automated tool for large-scale environmental monitoring in remote and hard-to-access Arctic regions, significantly reducing labor costs compared to traditional manual surveys.
- This study reveals a critical domain shift problem when the model is trained on one coastal area and tested on another (mAP50 drops from 0.692 to 0.432), highlighting the need for further research on model generalization and data augmentation strategies for operational Arctic monitoring.

**Abstract:**

Marine litter beach accumulation is a pressing issue not only for urban coastal areas but also for remote and uninhabited regions such as the Arctic. Existing manual litter collection and identification methods remain labor-intensive. An assessment of the volume and composition of marine litter was conducted for the Russian Arctic coastlines using high-resolution airphotos. The method is based on training the You Only Look Once (YOLOv8, 2023) convolutional neural network (CNN) in the Oriented Bounding Box (OBB) configuration to recognize plastic, metal, fishing gear, wood, and other types of marine litter on images. The best detection performance was obtained for metal and plastic—large, visually contrasting litter items. The worst performance was obtained for wood and other debris, primarily due to their similarity to the natural background. Machine learning (ML) methods combined with remote sensing data represent a new high-precision tool for large-scale environmental monitoring in the Arctic.

## 1. Introduction

Plastic pollution of the ocean has become a global environmental problem. Marine litter beach accumulation is a pressing issue not only for urban coastal areas but also for remote and uninhabited regions such as the Arctic [1]. The Arctic zone of the Russian Federation (AZRF is a key center of the country’s mineral resource complex. However, the unique and highly vulnerable natural environment of the Arctic is being disturbed by human activity [2,3]. Marine litter has begun to appear in a region that has long been considered pristine.

According to the United Nations Environment Programme (UNEP), marine litter is any persistent, manufactured or processed solid material discarded into the sea or rivers or on beaches; brought indirectly to the sea with rivers, sewage, storm water, or winds; or discarded or lost at sea [4]. Marine litter monitoring has been carried out by the Russian State Hydrometeorological University since 2018, including in the Arctic as part of annual scientific research expeditions in the framework of the Arctic Floating University Programme (AFU) [5]. Manual marine litter collection has an important advantage: it allows the type of material and its origin to be accurately determined, and it covers a wide range of object sizes (down to 2.5 cm). However, manual collection and identification of litter remain labor-intensive and result in high costs.

Remote sensing technologies have become a reliable tool for environmental monitoring. Specialized spectral indices, such as the Floating Debris Index (FDI) [6] and the Plastic Index (PI) [7], have been developed to detect plastics from satellite imagery. The spectral properties of plastics make it possible to distinguish them from the background in the Near-Infrared (NIR) and Short-Wave Infrared (SWIR) spectral bands [8]. However, satellite methods have a number of limitations in detecting marine litter related to spatial resolution and survey conditions [9]. The use of unmanned aerial systems helps to overcome these limitations, as the survey altitude is significantly lower than that of satellites. UAS have previously proven to be effective in the development and operation of mineral deposits [10,11], including monitoring the activities of oil industry facilities [12], assessing the effectiveness of land reclamation [13], as well as forest monitoring [14] and green-space monitoring in cities [15].

Recently, remote sensing and machine learning methods have been successfully adapted for monitoring marine litter floating on the water’s surface [16,17], and marine litter washed ashore [18,19]. Existing studies have demonstrated the potential of deep learning, including the YOLO model, for marine litter detection [20,21]. However, they are often limited to a single type of litter, typically plastic. Random data splitting can lead to overly optimistic performance estimates, since images from the same locations may appear in both the training and test sets. In Russia, the Clean Shore project demonstrated the potential of neural networks for litter detection in coastal areas [22]. A system was developed for ship-based camera imagery to detect litter, birds, and water surface artifacts [23].

However, these approaches are not directly transferable to UAS-based surveys of Arctic beaches. Arctic coastlines present specific difficulties: objects are small relative to the frame, background changes in weather and lighting, and natural items such as wood, stones, and algae resemble litter. These factors create a need for methods specifically adapted to Arctic features.

In order to develop an alternative approach to environmental monitoring in the Arctic, aerial photography of three coasts was conducted for the first time in 2023 as part of the expedition of the Arctic Floating University. The obtained materials were used to train a convolutional neural network to recognize various types of marine litter. The authors created their own dataset containing anthropogenic waste present in the images.

The objectives of this study were to:

1. Assess the feasibility of using the YOLOv8s-OBB model for automated detection of marine litter on Arctic beaches;
2. Evaluate the model’s performance in terms of precision, recall, and mean Average Precision (mAP) across five litter categories: metal, plastic, fishing gear, wood, and other;
3. Compare the results of automated detection with those obtained through manual collection and visual interpretation of unmanned aerial vehicle (UAV) imagery.

We tested the following hypotheses:

1. The YOLOv8-OBB model can detect marine litter on Arctic beaches;
2. The performance of the model varies depending on the type of litter, with visually distinct materials (metal and plastic) being detected more accurately than materials that blend in with the natural background;
3. The domain shift split approach, which separates training and test data geographically, provides a more realistic assessment of the generalization capability of the model compared to a random split.

## 2. Materials and Methods

### 2.1. Manual Collection and Identification of Marine Litter on Arctic Beaches in 2023

The Novaya Zemlya archipelago is a site of Russian regular monitoring of beached marine macrolitter (>2.5 cm) in the Barents and Kara Sea regions. Since 2018, the litter has been collected on several beaches from the northernmost part of the Northern (Severny) island during the summer expeditions of the AFU [24] according to the OSPAR (North-Atlantic region Convention) methodology [25] and recommendations of the AMAP (Arctic Monitoring and Assessment Programme) [26]. All items collected from a 100 m long beach strip were classified using the MSFD codes, dividing all litter into several categories, artificial polymers, metal, glass, rubber, textiles, etc. [27]; counted; and weighted.

**Figure 1:**
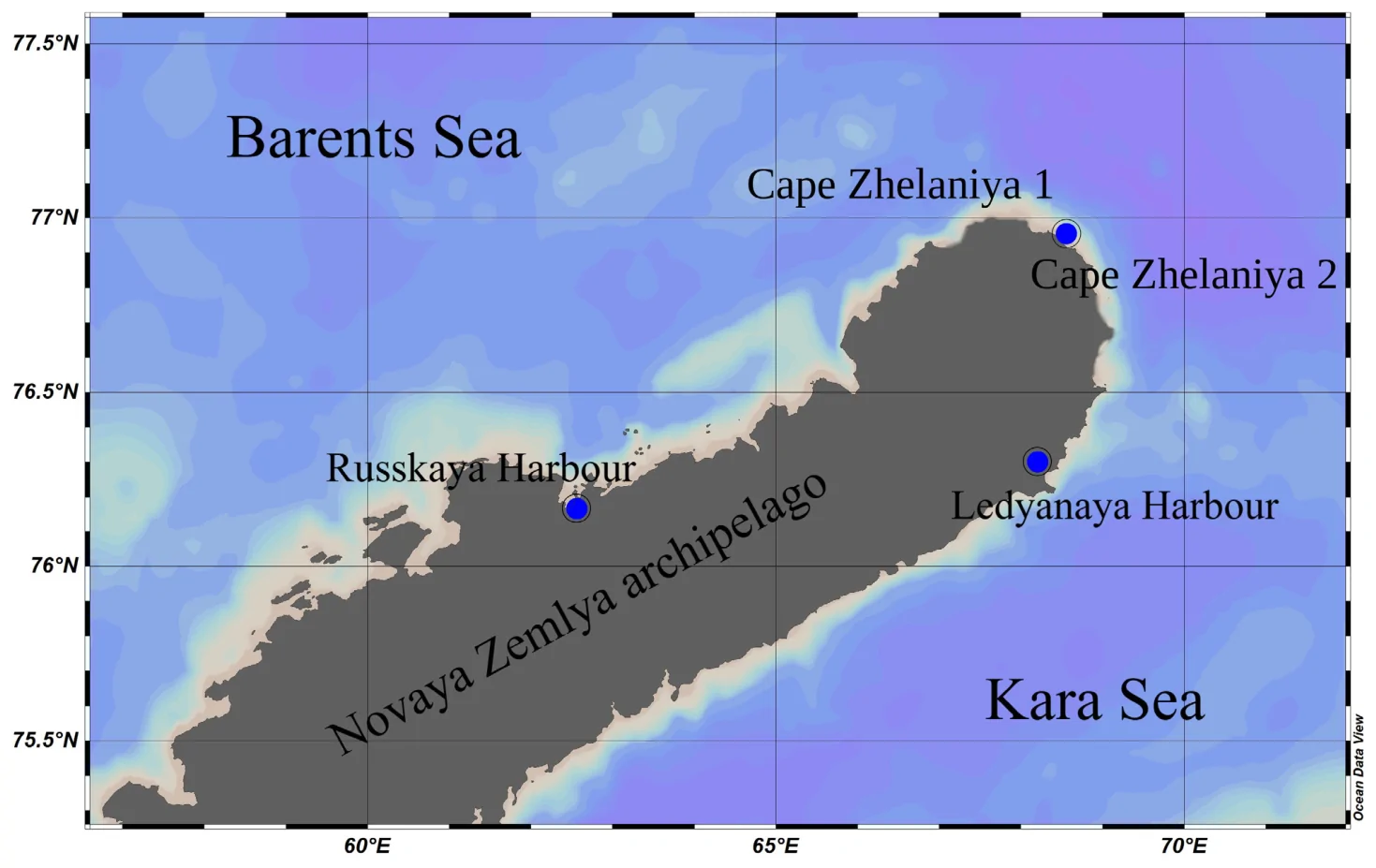
Map of the Novaya Zemlya archipelago with the marine litter monitoring sites in the framework of the Arctic Floating University cruise in 2023.

In July 2023, three beaches were surveyed: Russkaya Harbour (RH), Cape Zhelaniya (CZ), and Ledyanaya Harbour (LH). Seven 100 m long strips of the beach (3: RH, 2: CZ, and 2: LH) were successively surveyed by a team of volunteers guided by a professional team of scientists. A map of the Novaya Zemlya archipelago with the marine litter monitoring sites is presented in Figure 1.

### 2.2. Aerial Beach Survey and Photogrammetric Processing of Aerial Images

In 2023, manual litter collection on the beaches of Novaya Zemlya was held in parallel with aerial imaging for the first time. A survey of the coastal area was conducted using a DJI Mavic Air 2 quadcopter at flight altitudes of 20, 40, 50, and 100 m. The beach length was up to 1 km. The imagery was captured in nadir view along the shoreline in both manual and automatic modes based on a pre-determined flight mission. The capture rate was 1 frame per second at a flight speed of 2 m/s. The image frame size was 4000 × 3000 pixels. The forward overlap exceeded 70%. A total of 1752 images were collected. The survey parameters are presented in Table 1 [28].

The acquired images (1752 in total) were then processed using Agisoft Metashape Professional software v.2.2.2. For each survey site, a digital surface model (DSM) was generated from the dense point cloud. The DSMs were subsequently used to produce six orthophotomaps (one per flight altitude; see Table 1)—high-resolution, geometrically corrected planimetric representations of the terrain. This step was essential to eliminate the processing of overlapping image footprints, ensuring that each ground area was represented only once in the subsequent analysis [29].

### 2.3. Manual Image Screening and Selection of Object Classes

At the next stage, the authors performed manual image screening (visual interpretation) for all images acquired during the survey. Based on the results, anthropogenic objects were counted and recorded manually in protocols with assignment of the corresponding class. This method proved to be labor-intensive and fairly subjective; therefore, it was decided to optimize the process and reduce the influence of operator error through automated image processing using machine learning [28].

The results of the preliminary visual data analysis show that the main categories of marine litter on Arctic shores include plastic items, metal, fishing gear, and wood. These categories were assigned to the corresponding classes. Items that could not be clearly identified were placed in the “Other” category. Information on these classes is presented in Table 2.

### 2.4. Selection of the Machine Learning Model

A machine learning model was selected based on a review of the available scientific literature. The most effective supervised learning methods are Random Forest (classification accuracy: 92–98%) and Support Vector Machine (89–100%) [30], while the most effective unsupervised learning method is K-means (up to 81.4%) [31]. A new stage in machine learning is deep learning, and the use of convolutional neural networks for the detection of litter image items in particular. CNNs are a specialized class of artificial neural networks designed to process data with spatial structure, such as images and videos [32]. All modern image detection algorithms can be divided into two broad categories: two-stage detectors and one-stage detectors. One-stage models are characterized by high inference speed and simplicity of architecture, making them the preferred choice for this task [33].

One of the most widespread one-stage CNNs is YOLO, a family of one-stage image detectors that process images in a single pass through a convolutional neural network. Several versions of YOLO have been developed to date [34]. The YOLOv8s (small) 2023 model was selected in the Oriented Bounding Box configuration. This version of YOLOv8 was chosen as a compromise between accuracy and computational efficiency. OBB allows for a more accurate description of objects located at arbitrary angles to the image axes, as it includes a rotation angle and 8 coordinates of the labeled rectangles instead of 4 [35]. This is particularly important for marine litter, as many litter items, such as fishing nets, ropes, wooden planks, and irregular plastic fragments, are not aligned with the image axis and exhibit arbitrary orientations. Processing time increases due to the larger number of coordinates. However, this does not introduce any specific errors inherent in the OBB format. Other human-related errors may still occur, such as incorrect data export, selection of a model that does not support the OBB, etc.

### 2.5. Data Labeling and Dataset Preparation

For anthropogenic waste detection on images, several published datasets can be used containing images of marine litter on various surfaces: underwater [36], on the water’s surface [37], on a white surface [38], and on land and beaches [39]. The datasets were not applicable within the framework of this study for the following reasons:

1. Different flight altitudes;
2. Absence of typical Arctic litter items (e.g., metal barrels and fishing gear);
3. The underlying surface differs from Arctic beaches: sand instead of pebbles and large stones, typical for the Arctic.

In this regard, we prepared our own dataset unique to the conditions of Arctic shores. To create our dataset, the Label Studio data labeling and annotation system was used. The initial data consisted of image patches from orthophotos obtained at previous stages. Dataset labeling was performed manually. The minimum detectable size of litter item depended on flight altitude. Given the sensor resolution of 4000 × 3000 pixels and focal length of 4.0 mm, the Ground Sampling Distance (GSD) was approximately 0.8 cm/pixel at a 20 m altitude. The GSD increased to 1.6 cm/pixel at 40 m, 2.0 cm/pixel at 50 m, and 4.0 cm/pixel at 100 m. Assuming a minimum object size range of 10–15 pixels, this corresponded to detectable litter item sizes in ranges of approximately 8–12 cm at 20 m, 16–24 cm at 40 m, 20–30 cm at 50 m, and 40–60 cm at 100 m.

A separate annotation was created for each litter item. For partially buried items, only the visible part was labeled. The bounding box orientation remained unchanged for overlapping objects annotated by their visible parts.

The main difficulties encountered during data labeling were related to:

1. Overlapping of litter items (e.g., fishing net on a metal barrel);
2. Complex non-linear shapes of litter items, such as fishing nets;
3. Inability to accurately determine the type of litter item from aerial photos;
4. Uneven stitching in orthophotos.

After the completion of labeling, the data were exported in the YOLOv8sOBB (TXT) format. Each bounding box is described by a set of parameters: x, y, w, h, and θ (x, y—item center coordinates; w, h—box dimensions; θ—rotation angle relative to the horizontal axis). Subsequently, for model training, the original images of 4000 × 3000 pixels were resized to the YOLO library format—squares of 1024 × 1024 pixels in JPEG—using the SAHI library. In total, from the original 1752 UAV images, 1039 square images with a size of 1024 × 1024 pixels were obtained. The total number of labeled litter items was 1973. No data loss occurred during the format conversion process.

**Figure 2:**
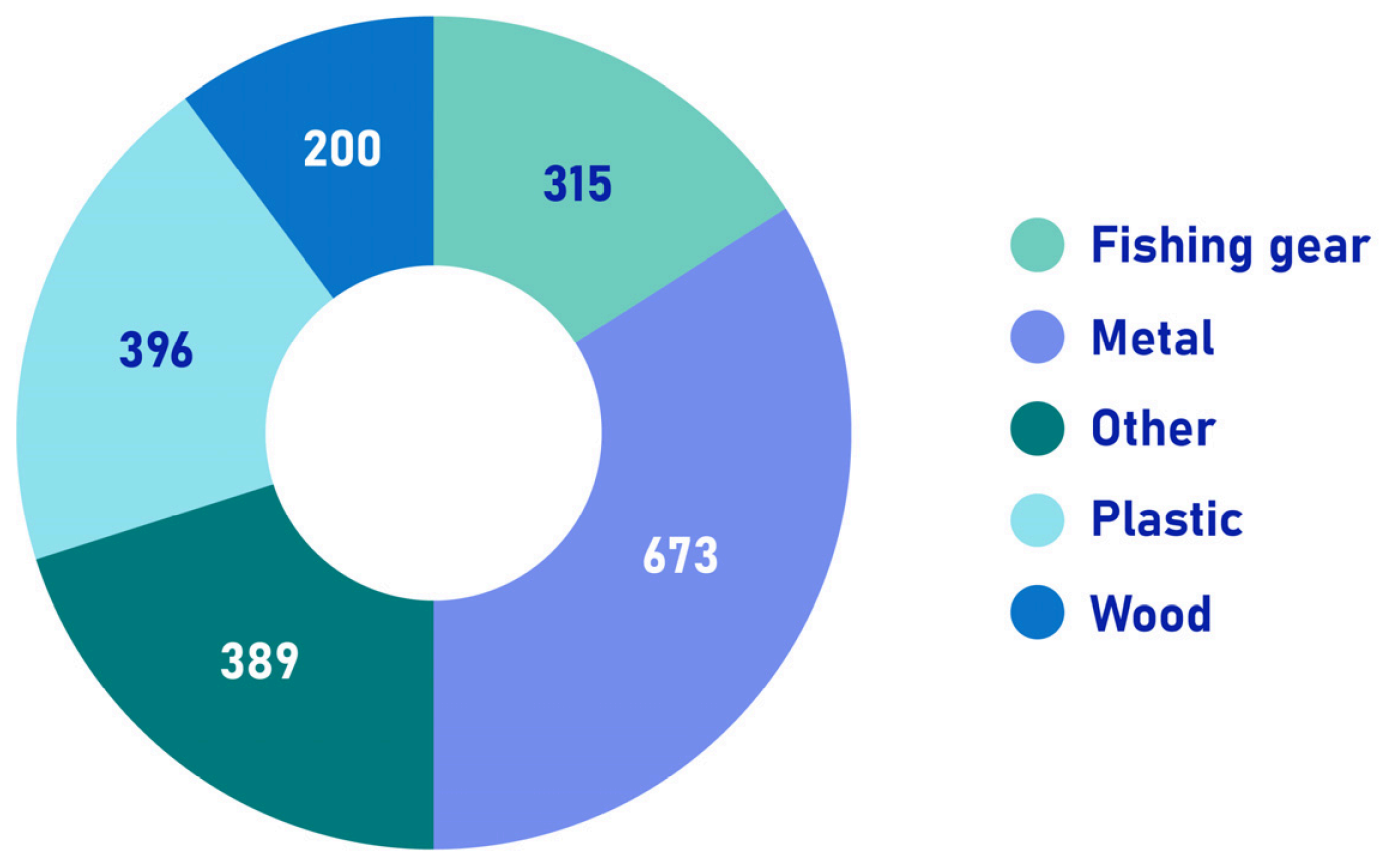
Distribution of the number of dataset litter items by class.

This indicates the low density of items on the aerial photos. This feature is characteristic of marine litter datasets and represents a major problem in model training [40]. Class imbalance was also identified: the smallest number of litter items was in the “Wood” class (10%) and the largest in the “Metal” class (34%). To address this shortcoming, built-in geometric, compositional, color, and special augmentations of YOLOv8s OBB were used (image rotation, horizontal and vertical flipping, image scaling, image mosaic, etc.) [41]. The distribution diagram of the number of litter items by class is presented in Figure 2.

### 2.6. Model Training

To train the model, the original dataset was divided into three subsets: training set, validation set, and test set. The dataset was split using two approaches: random split and domain shift split.

Random split. In the first approach, 1039 images were randomly divided in a 70/20/10 proportion: 727, 208, and 104 images for train, val, and test, respectively.

Domain Shift Split. In the second approach, the dataset was split geographically: images from Russkaya Harbour were assigned to the training and validation sets. Images from Cape Zhelaniya and Ledyanaya Harbour were reserved exclusively for testing. This resulted in 651, 284, and 104 images for training, validation, and testing, respectively. This approach simulates a real-world scenario in which the model is trained on data from certain surveyed areas and evaluated on completely unseen locations, thereby assessing its generalization capability to new geographical regions.

Notably, the total number of images across all splits remained consistent with the overall dataset size (1039 images), ensuring that all annotated data were utilized while maintaining strict separation between training and test sets.

The training parameters were determined empirically. The number of epochs was set to 120 based on convergence analysis: validation metrics stabilized after a range of approximately 100–120 epochs, with only marginal improvements beyond that point. An early stopping mechanism with a patience of 50 epochs was applied to prevent overfitting and reduce computational cost. Alternative configurations (including 200 epochs and patience of 30) were tested, but the selected settings provided the best trade-off between performance and efficiency. The input image size was 1024 × 1024 pixels, and the batch size was 8.

AdamW was used as the optimizer. The choice of the AdamW optimizer was driven by its key advantages in the context of sparse experimental data and limited sample size. The adaptive mechanism for adjusting the learning rate for each parameter enabled efficient handling of heterogeneous data, while the separate correction of L2 regularization helped improve the model’s generalization ability and reduce the risk of overfitting. An additional argument is AdamW’s robustness to the choice of initial learning rate, which simplifies the hyperparameter tuning process compared to alternative methods.

**Figure 3:**
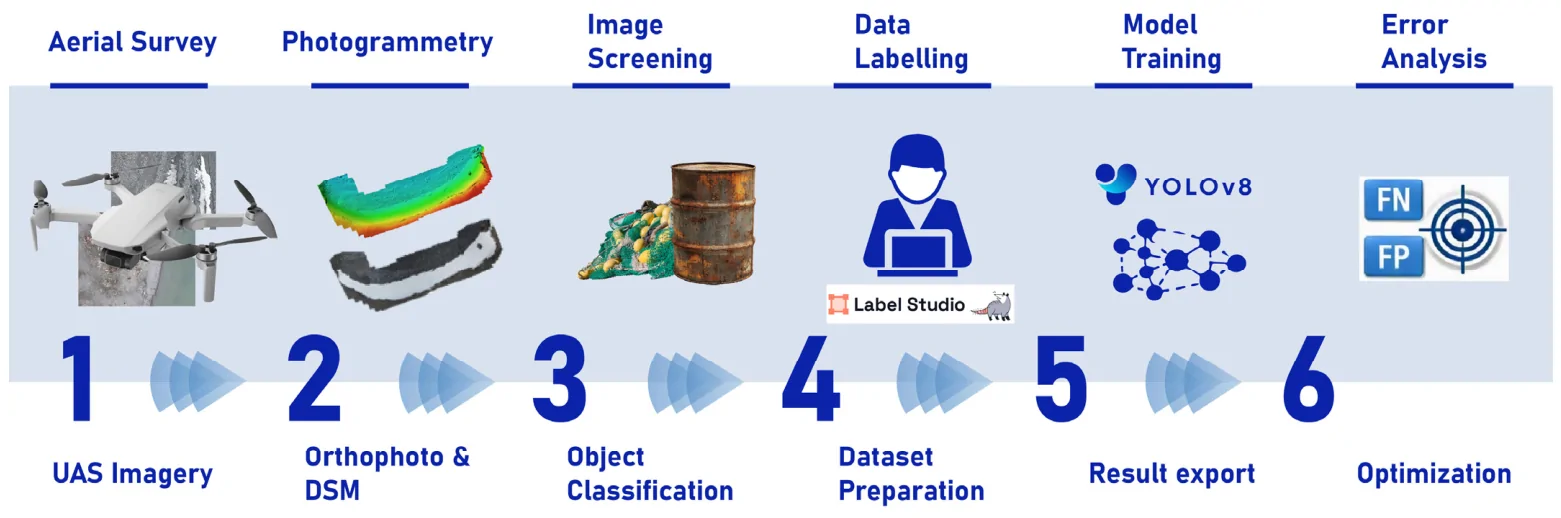
Methodological workflow for marine litter detection from aerial images using machine learning.

A complete methodological workflow, including all research stages described in Section 2, is presented in Figure 3.

## 3. Results

### 3.1. Manual Litter Collection Results in 2023

In 2023, the largest amount of marine litter was detected in the Russian Harbor and Cape Zhelaniya; the lowest was in Ledyanaya Harbor. On average, and as in previous years [24], the density of marine litter is higher on the Barents Sea side of the archipelago. A small fraction (<50 cm) of macrolitter dominates in most locations, with the most common litter items being fragments of fishing nets, parts of fishing crates, strapping bands, pieces of ropes, as well as various plastic containers (buckets, baskets, bottles, and food containers), lids, and small fragments of plastic. In total, 2188 litter items were detected and classified, collected from 700 m of the beach strip in total (seven 100 m strips on three beaches).

### 3.2. Training Dynamic

**Figure 4:**
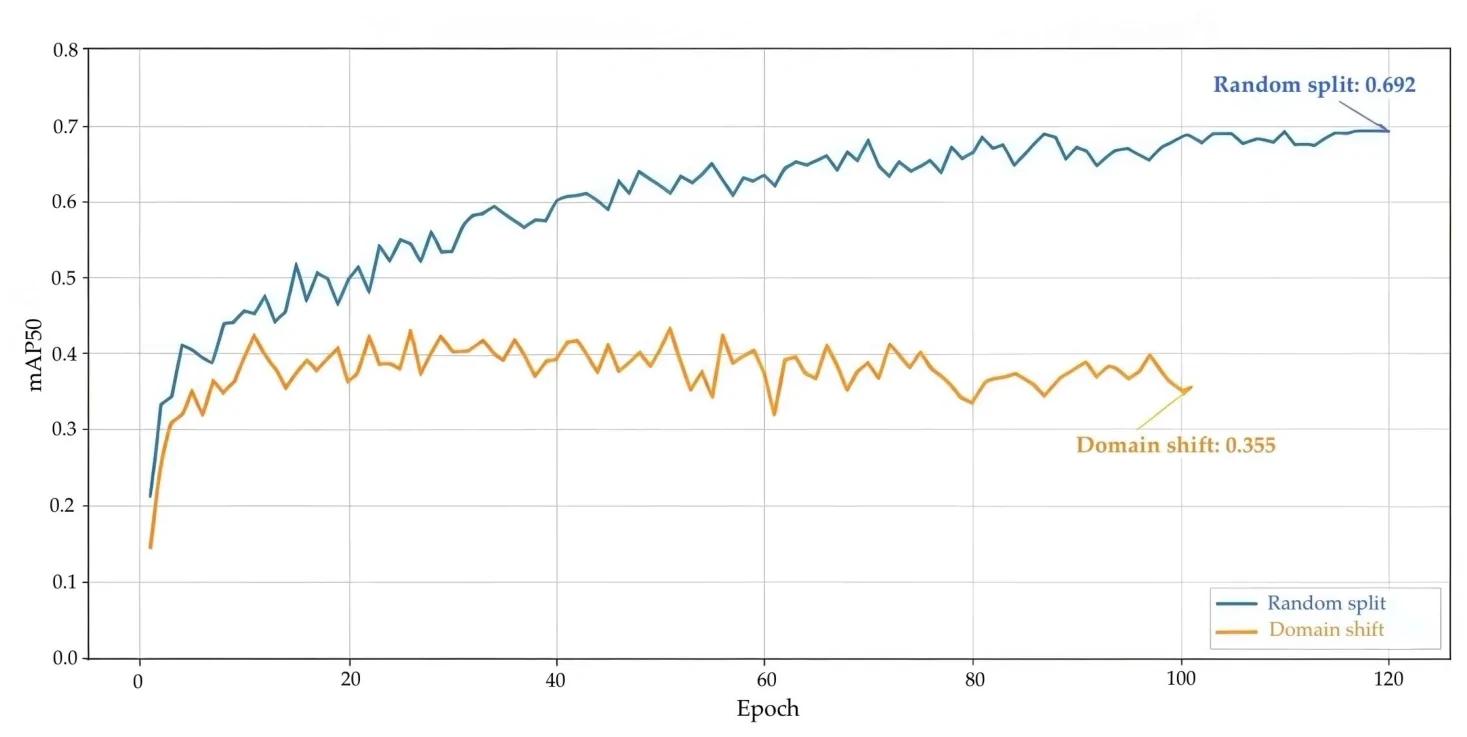
Differences in training on the two datasets.

**Figure 5:**
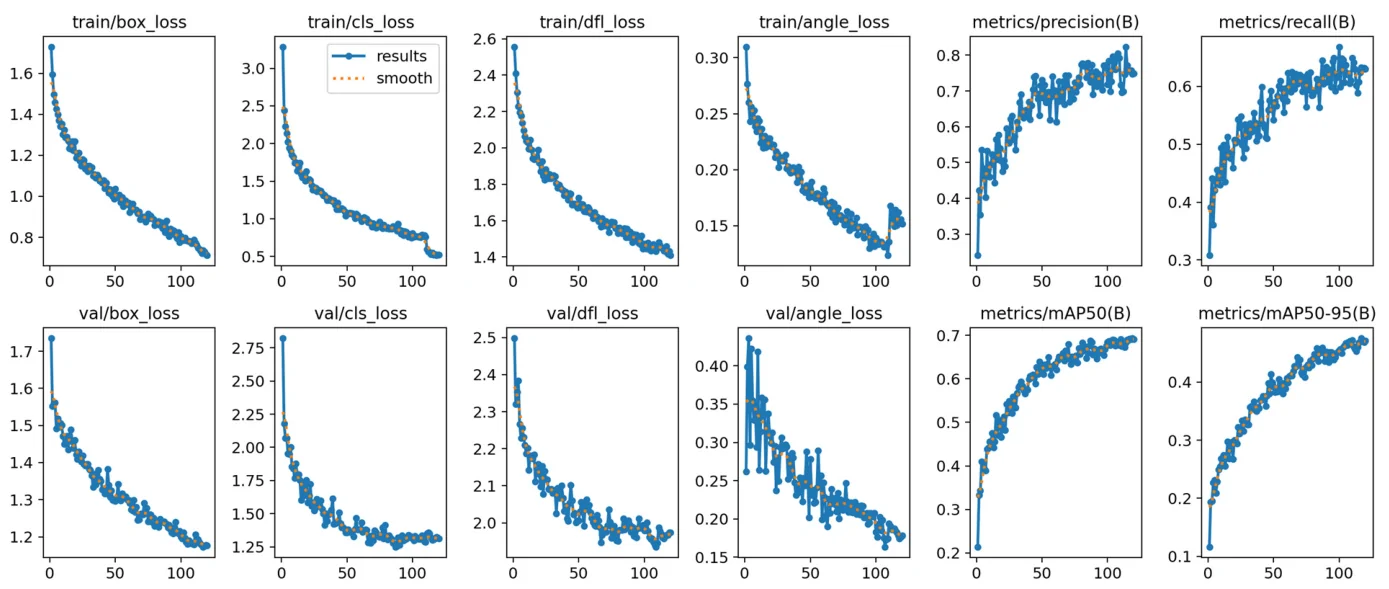
Training dynamics on the test and validation sets.

A comparative analysis of the training dynamics under different data split types revealed strong differences. Under the random split, a rapid increase in mAP50 was observed during the first 40 epochs, followed by a slowdown and plateau after 70 epochs. The validation loss decreased synchronously with the training loss, with no overfitting observed. Under the domain shift split, the training dynamic was completely different: the model did not achieve an acceptable level of accuracy, with key performance metrics fluctuating and showing no sustained growth. At the end of training, metrics declined while the training loss continued to decrease and the validation loss increased. This divergence is a classic sign of overfitting (Figure 4 and Figure 5).

### 3.3. Visualization of Results

A final model was obtained after training. For a new input image, the model produces the following output:

- The coordinates of the bounding boxes where plastic is presumably located;
- The confidence level for each detection;
- The class of the identified litter item.

**Figure 6:**
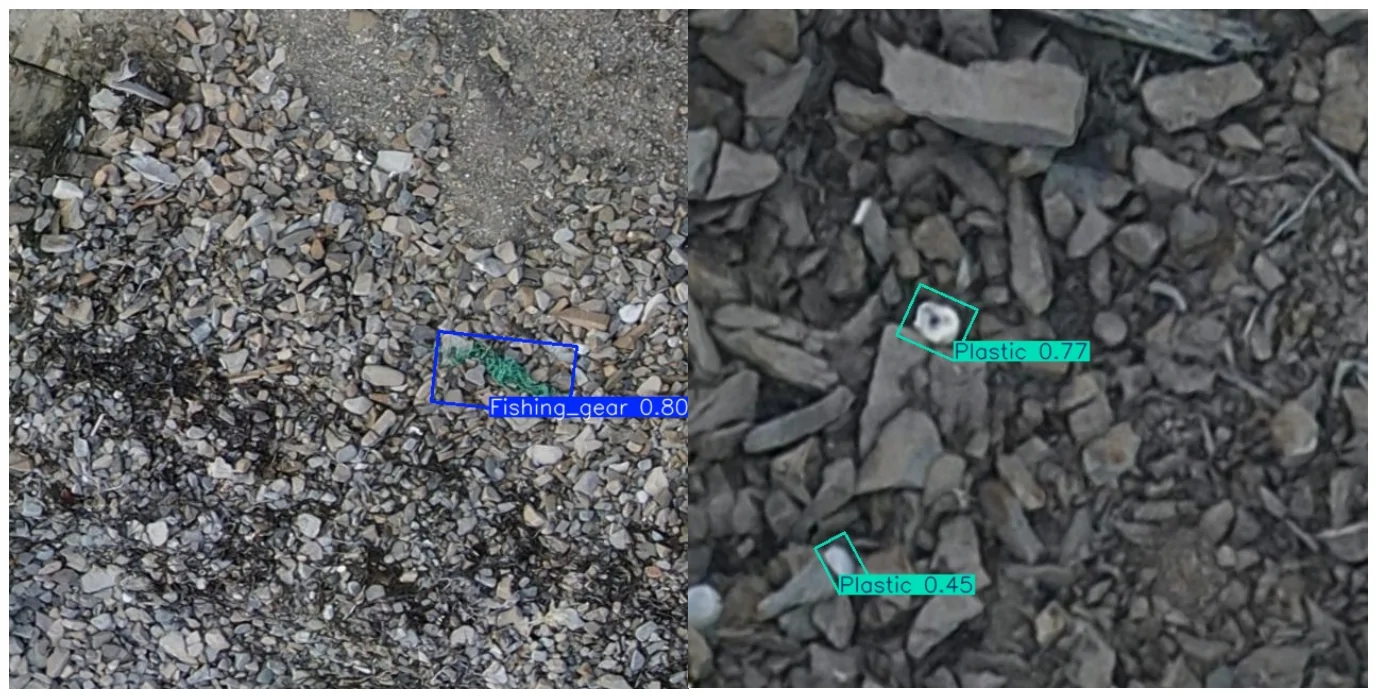
Example of detection for the “Fishing_gear” and “Plastic” classes.

**Figure 7:**
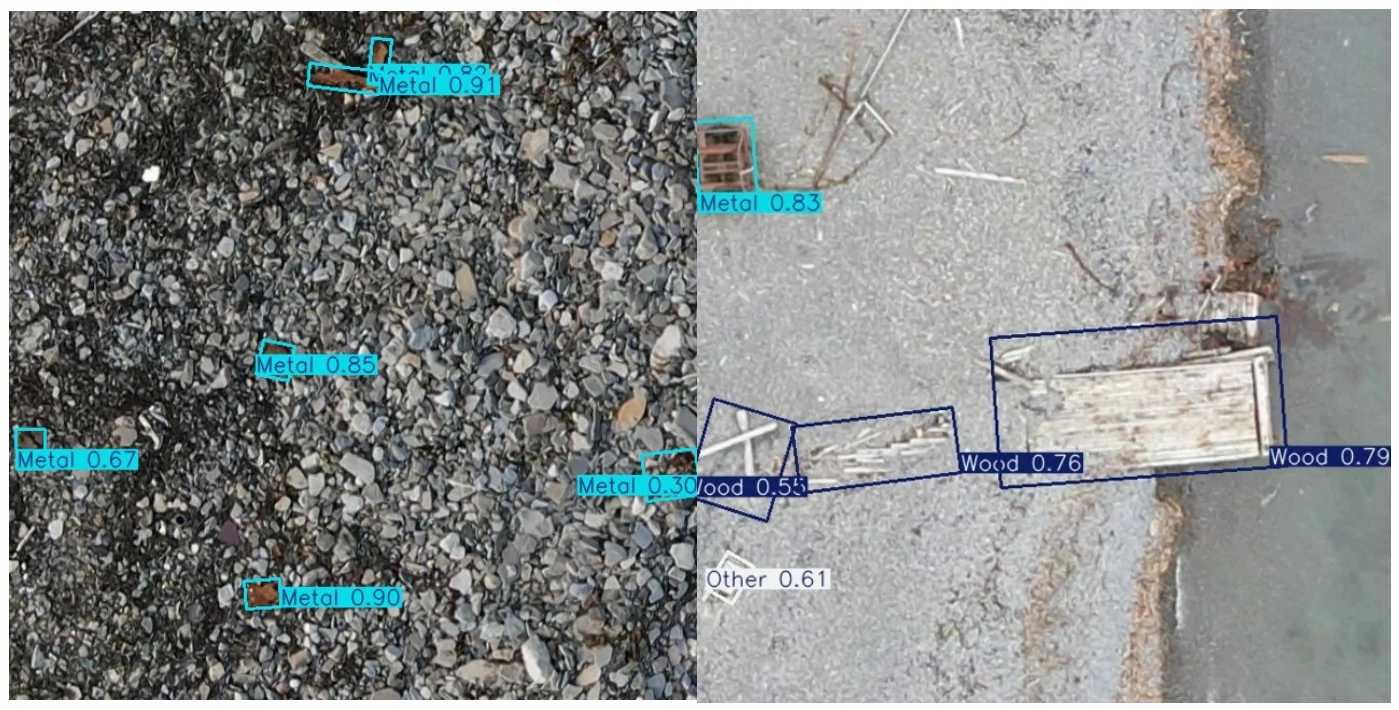
Example of detection for the “Metal”, “Wood”, and “Other” classes.

Examples of the model’s performance on the test data for different object classes are presented in Figure 6 and Figure 7. Low-confidence detections (e.g., 0.45 for plastic in Figure 6 and 0.3 for metal in Figure 7) indicate that the litter item resembles natural background features. The visual similarity to pebbles and stones may mislead the model, especially for litter items whose size is close to the detection limit (8–12 cm at a flight altitude of 20 m).

### 3.4. Model Performance Assessment

To assess the quality of model performance, the following metrics were used: precision, recall, mAP50, and mAP50-95. Precision is the proportion of correctly identified items among all items identified by the model. Recall is the proportion of correctly detected items among all actually existing target items. mAP (mean Average Precision) is the Average Precision averaged over all classes. The priority metric is recall, since within the framework of this study, missing actual litter causes greater environmental damage than a false-positive detection in the context of Arctic territories. The results of model performance on validation and test sets are presented in Table 3 and Table 4, and on the domain-shifted split in Table 5.

The analysis of the model performance results shows that the difficulty of detecting different classes varies. The criteria for evaluating the success of the experiment were based on the following metrics: mAP50 ≥ 0.65, mAP50-95 ≥ 0.35, precision ≥ 0.70, and recall ≥ 0.55 to each class [42]. These thresholds were applied individually to each class, rather than as an overall average, to avoid masking poor performance in specific categories. Under the random split, three classes met the criteria: plastic, fishing gear, and metal. However, two classes, other and wood, did not reach the required thresholds. This worse performance can be attributed to the limited number of training examples and their similarity to the natural background.

The model recognizes metallic litter most accurately, which can be attributed to both its abundance in the training data and its unique visual characteristics. Metallic items typically have a characteristic brownish-metallic shine and clear, well-defined geometric shapes.

Plastic litter, such as bottles, bags, and boxes, has high detection accuracy thanks to its bright coloration. The model recognizes it easily. However, its variety of shapes and shades can make detection more challenging. Fishing gear, like nets, ropes, and cords, is more difficult for the model to recognize because of the heterogeneity of its structure and tangled shape. Nevertheless, bright colors help with detection.

The “Other” category has a low detection accuracy, as the model misses approximately half of the items in this category. This is due to the internal diversity of the “Other” group, which includes a variety of visually different items that do not fit well into the other four categories. Wood (planks, boxes, and driftwood) has the worst performance in terms of recognition across all metrics, due to its visual similarity to natural objects.

The performance difference between the two splitting methods can be explained by the fundamental differences in the way the data are distributed. In the random split method, images from all beaches are randomly divided into training, validation, and test sets. This may lead to the testing images being from the same geographic areas as the training data, which can cause the model to learn location-specific features and overestimate performance metrics. In contrast, the domain shift split method ensures that there is a strict separation between the training and testing data, with no overlapping survey locations.

### 3.5. Error Analysis of Model Performance

**Figure 8:**
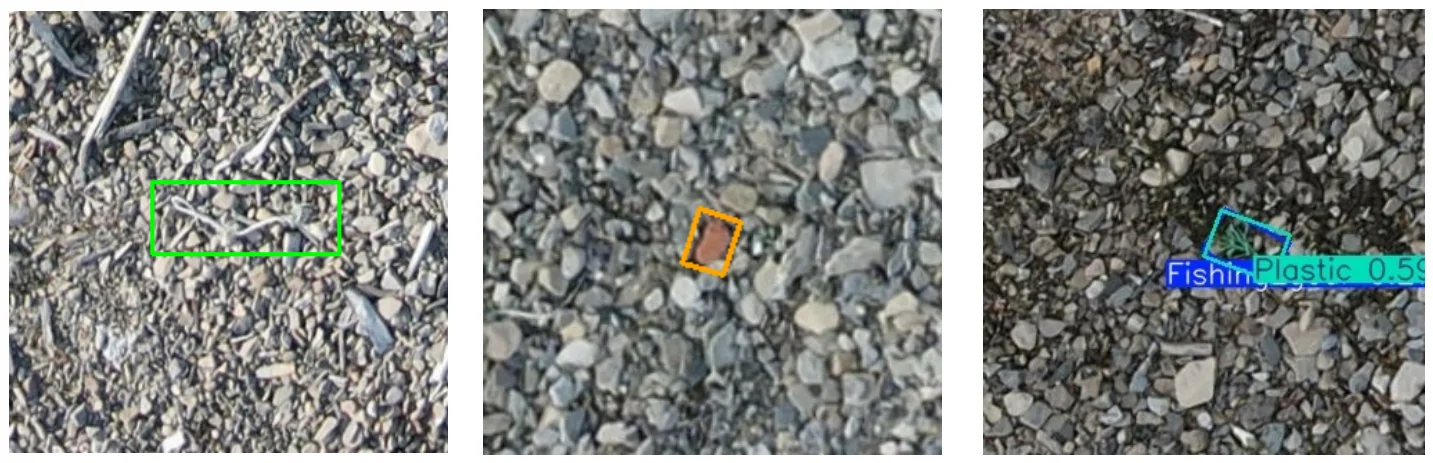
Examples of detection errors: FN: a fishing net missed by the model (green bounding box), FP: a stone misclassified as metal (orange bounding box), and WrongClass: fishing net incorrectly identified as plastic.

The model predictions were compared with the original annotations at the final stage. Each litter item was classified by error type: FN (false negative: missed item), FP (false positive: false alarm), and WrongClass: incorrect classification (Figure 8).

Table 6 presents the distribution of errors by type committed by the model. The distribution of errors by class is shown in Table 7. The confusion matrix shows the classification results of the YOLOv8s-OBB model. The rows represent the predicted classes and the columns represent the true classes. The the diagonal cells show correctly classified objects, and the off-diagonal cells show WrongClass. The last row (background) shows FN. The last column (background) shows FP.

**Figure 9:**
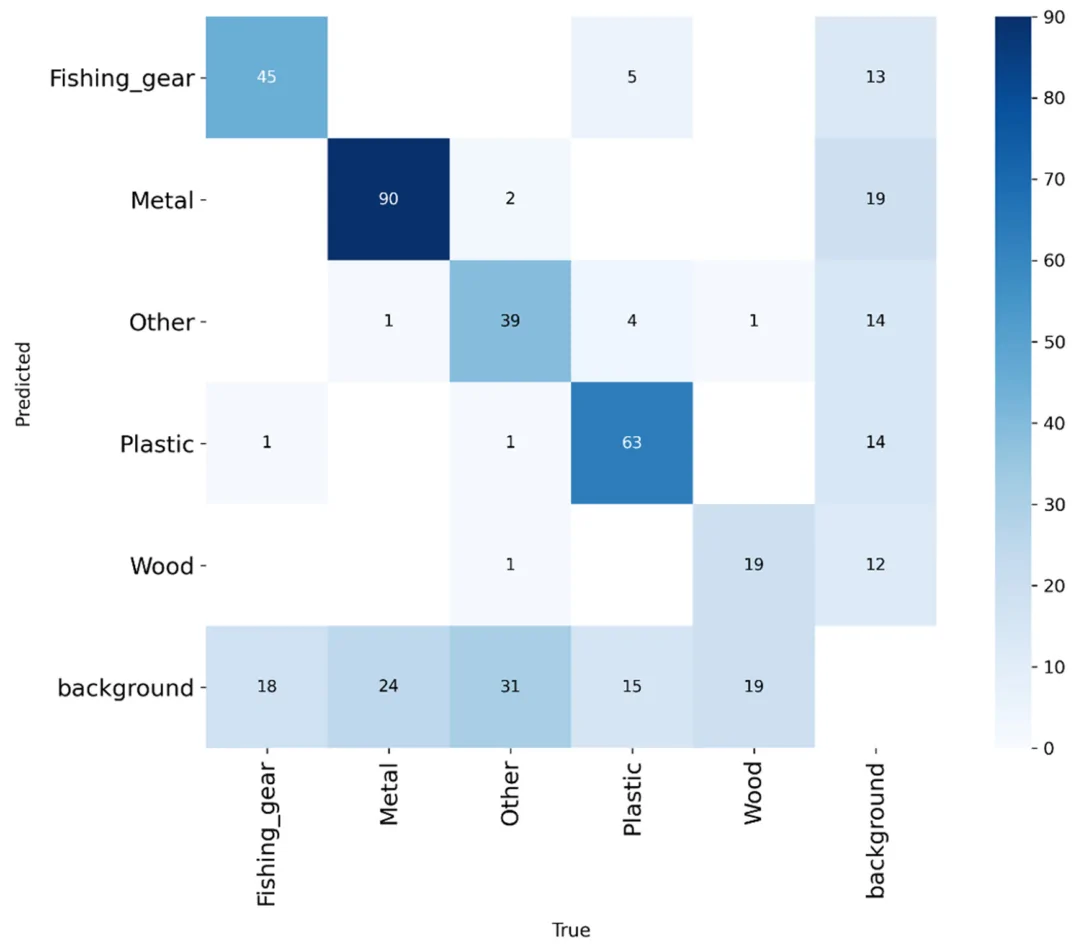
Confusion matrix.

The confusion matrix is presented in Figure 9.

Analysis of the model performance results on the validation set showed that there was virtually no confusion between classes (WrongClass accounted for only 8% of the total number of errors). However, the major error occurred precisely in object detection (FN and FP together accounted for 92%), rather than in assigning it to a particular class.

“Fishing_gear” and “Other” were most often mistakenly classified as “Plastic”. The highest number of misses (FNs) was characteristic of the “Other” class. This indicates either the need to subdivide objects within this class into additional categories or the need to expand the training set. At the same time, the model rarely misclassified extraneous litter items as “Other”, but it often missed actual litter items in this category. The highest number of false positives (FPs) was observed for the “Metal” class. Nevertheless, the model demonstrated a high detection rate for this class in the aerial images (Table 3 and Table 4).

## 4. Discussion

The results of automated detection of marine litter on aerial photographs using machine learning. This study was the first to perform an automated detection of marine litter on Arctic coasts using the YOLOv8-OBB convolutional neural network. The main limitation when working with aerial photos is the detection threshold, which depends on flight altitude. The minimum detectable litter item size varied with flight altitude: at 20 m: 8–12 cm; at 40 m: 16–24 cm; at 50 m: 20–30 cm; and at 100 m: 40–60 cm. In addition to the size, the possibility of detecting marine litter is influenced by the color and shape of the item: with a bright contrast, background, and a clear geometric shape, the item is more likely to be identified. For example, the best detection results are typical for the “Metal” (mAP50 = 0.811) and “Plastic” (mAP50 = 0.789) classes, while the most challenging classes to recognize were “Wood” (mAP50 = 0.551) and “Other” (mAP50 = 0.556).

Domain shift. The risk of a phenomenon such as a domain shift was noted when statistical patterns identified by the model on one subset of data lose their significance for another. The domain shift is caused by the heterogeneity of the initial data: despite the fact that the survey was conducted in the same area, the studied areas had different types of background, such as pebbles, sand, and water, and were taken from different heights (20, 40, 50, 100 m). This paper presents the results of processing images of all beaches, without dividing into separate territories, which was necessary for training the model. As a result, the model may perform worse on other parts of the coast with new shooting parameters. To minimize the domain shift, further research assumes the use of data augmentation with simulations of various shooting conditions (changes in height, illumination, and background type), followed by further training of the model and evaluation of its generalizing ability on independent test sets.

Comparison of marine litter monitoring methods. During this study, litter detection was conducted using three different methods:

1. Manual collection and classification of marine litter (on seven 100 m transects of three beaches);
2. Manual screening of UAS images and manual counting of marine litter on aerial photos (for the entire coastline of three surveyed beaches: 3547 m in total);
3. Automated detection and counting of marine litter using the YOLOv8 convolutional neural network (for the entire coastline of three beaches).

A significant advantage of the manual monitoring method is the ability to identify items of any size, including relatively small items (>2.5 cm), and in some cases to determine their origin (by label). A comparative analysis shows that the number of litter items identified through manual collection significantly exceeds the data obtained by manual image screening and machine learning: 2188 litter items per 700 m (manual collection) vs. 1973 litter items per 3547 m (manual image screening). This is due to the lower resolution of imaging, able to fix only large and well-seen colored litter items. However, the manual method’s main limitations are time and labor force. While a manual survey of one or two 100 m beach strips (transects) took 2 to 3 h of work from a group of 5 to 8 volunteers, an aerial survey was completed by one person (and one drone) in less than an hour covering the entire beach length (up to 1000 m).

As for the methods of processing aerial photographs, manual image screening is a rather labor-intensive process and is characterized by a high degree of subjectivity: an item on the image may be missed by an operator or incorrectly assigned to a class. The use of machine learning methods makes it possible to automate object detection and minimize the number of errors through multi-stage model training [43].

## 5. Conclusions

Arctic beaches are remote and difficult to access. Their length is considerable, and plastic litter continues to accumulate along their shores. Manual surveys are costly and logistically challenging. Remote sensing offers a clear advantage for assessing the overall volume of marine litter. It reliably identifies large items of litter starting from a 8–12 cm range in size, like fishing gear, crates, fish boxes, barrels, etc., which account for the majority of the total litter mass. Furthermore, the integration of machine learning into the processing pipeline helps to automate and accelerate the analysis of UAV imagery.

Automated detection of marine litter on Arctic beaches was performed based on aerial photography data using the YOLOv8s convolutional neural network in the Oriented Bounding Box configuration. A unique dataset was created to adapt the model to Arctic conditions. This study confirmed that the proposed approach is effective for detecting marine litter in the Arctic.

The main conclusions are:

1. Metal (673 items, 34.1%) and plastic (396 items, 20.1%) dominated the detected litter. Fishing gear also formed a substantial share (315 items, 16.0%).
2. Litter accumulation was uneven across the studied beaches. The highest density was recorded at Cape Zhelaniya (Barents Sea side), and the lowest at Ledyanaya Harbour. This pattern reflects the distance from major shipping routes and the level of anthropogenic impact.

A comparison of monitoring methods showed that manual collection provides the most detailed qualitative information and can be used to verify remote sensing results. However, it is limited to 100 m transects. UAV imagery covers larger areas but requires automated processing, as manual image review is labor-intensive and subjective. The proposed method is less accurate for small items but enables automated processing of large data volumes.

The most effective approach today is a combination of manual surveys and remote sensing. Manual collection remains essential for ground-truthing, while automated processing provides a clear picture of litter types, sizes, quantities, and spatial distribution. Given the growing anthropogenic pressure on aquatic ecosystems, environmental monitoring of coastal areas must become not only mandatory but also optimized.

This study confirmed three hypotheses:

1. The YOLOv8-OBB model can detect marine litter on Arctic beaches.
2. Detection performance varies by litter type, with metal and plastic being recognized more accurately than wood and other items that blend into the natural background.
3. A domain shift split provides a more realistic assessment of generalization than a random split, as it ensures that training and testing data come from geographically distinct areas.

Further research will be aimed at minimizing the impact of the domain shift by expanding the training sample through the use of data augmentation methods, including variations in shooting height, illumination angle, and background spectral characteristics, as well as using domain adaptation techniques such as fine tuning a pre-trained model based on data from new coast sites.

## Figures and Tables

**Table 1:** Parameters of aerial photography of Novaya Zemlya archipelago beaches using UAVs [28].

Survey Area	Flight Altitude, m	Number of Images	Effective Swath Width, m	Survey Area Length, m
Russkaya Harbour	40	414	37–40	770
	100	631		
Ledyanaya Harbour	20	87	27	337
	50	133	61	1002
Cape Zhelaniya (Barents Sea)	20	270	22	718
Cape Zhelaniya (Kara Sea)	20	217	16–24	720
Total	–	1752	–	3547

**Table 2:** Description of the training set classes.

Class Name	Code According to Classifier *	Class Description	Example Image from Airphoto
Plastic	G16, G7, G8, G9, G57, G79, G80	Jerry cans (square plastic containers with handle)/drink bottles ≤ 0.5 L/drink bottles > 0.5 L/cleaner bottles and containers/plastic fish boxes/plastic pieces	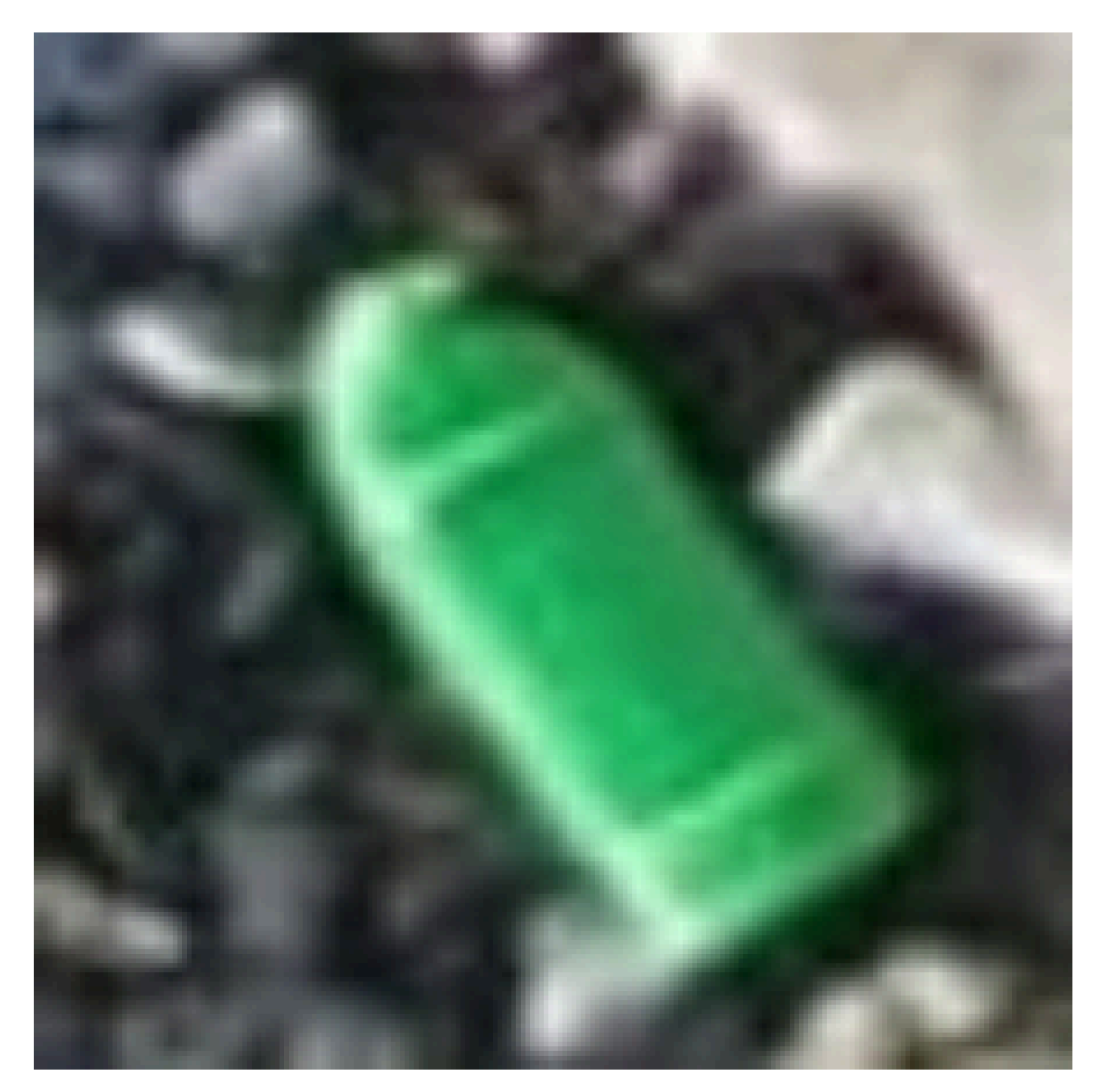
Fishing_gear	G49, G50, G53, G54, G56	Rope (diameter more than 1 cm)/string and cord (diameter less than 1 cm)/nets and pieces of net < 50 cm/nets and pieces of net > 50 cm/tangled nets/cord	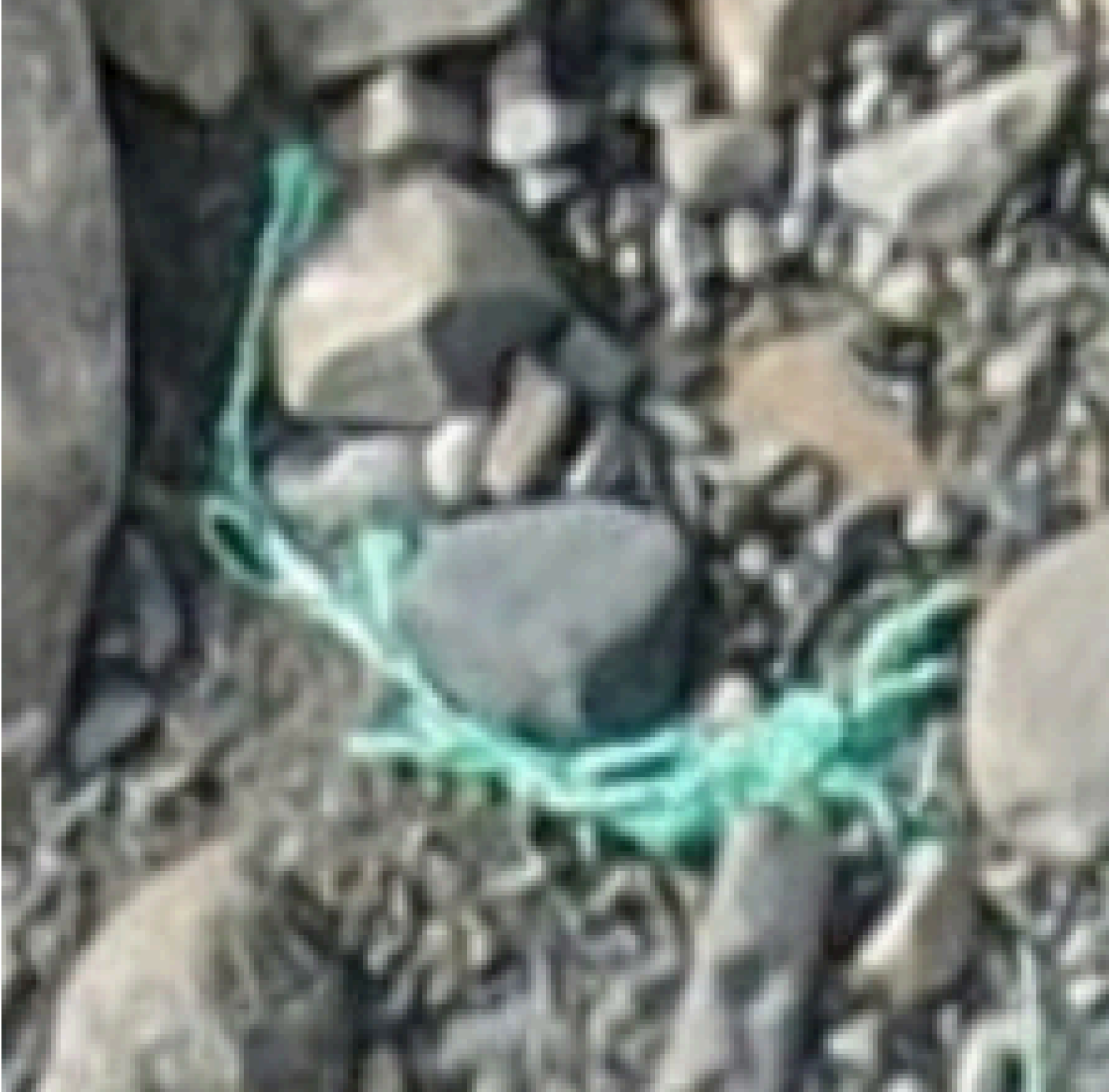
Metal	G187, G186, G198, G199	Drums, e.g., oil/industrial scrap/other metal pieces < 50 cm/other metal pieces > 50 cm	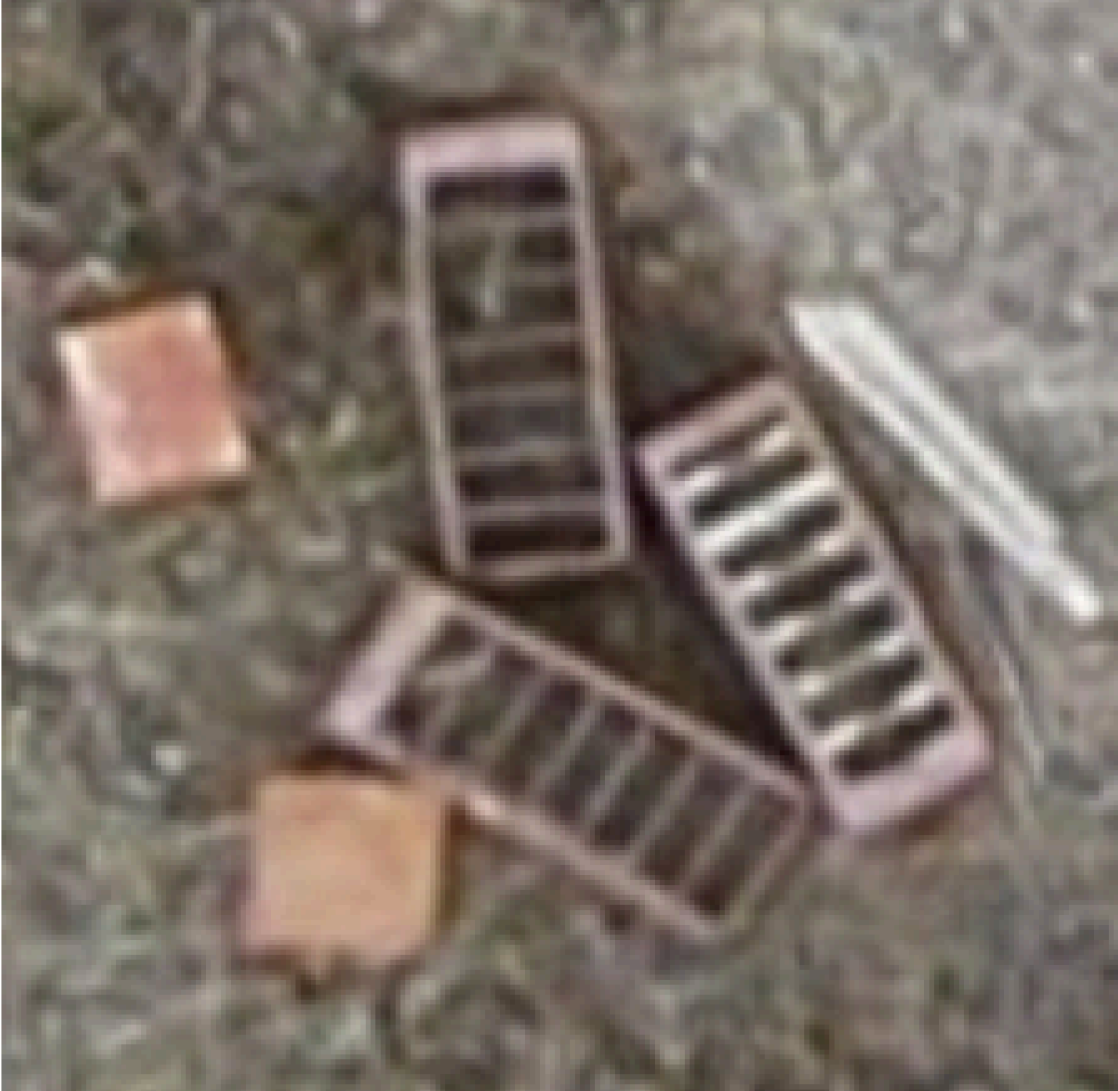
Wood	G164, G160, G162, G171, G172	Fish boxes/pallets/crates/other wood < 50 cm/other wood > 50 cm	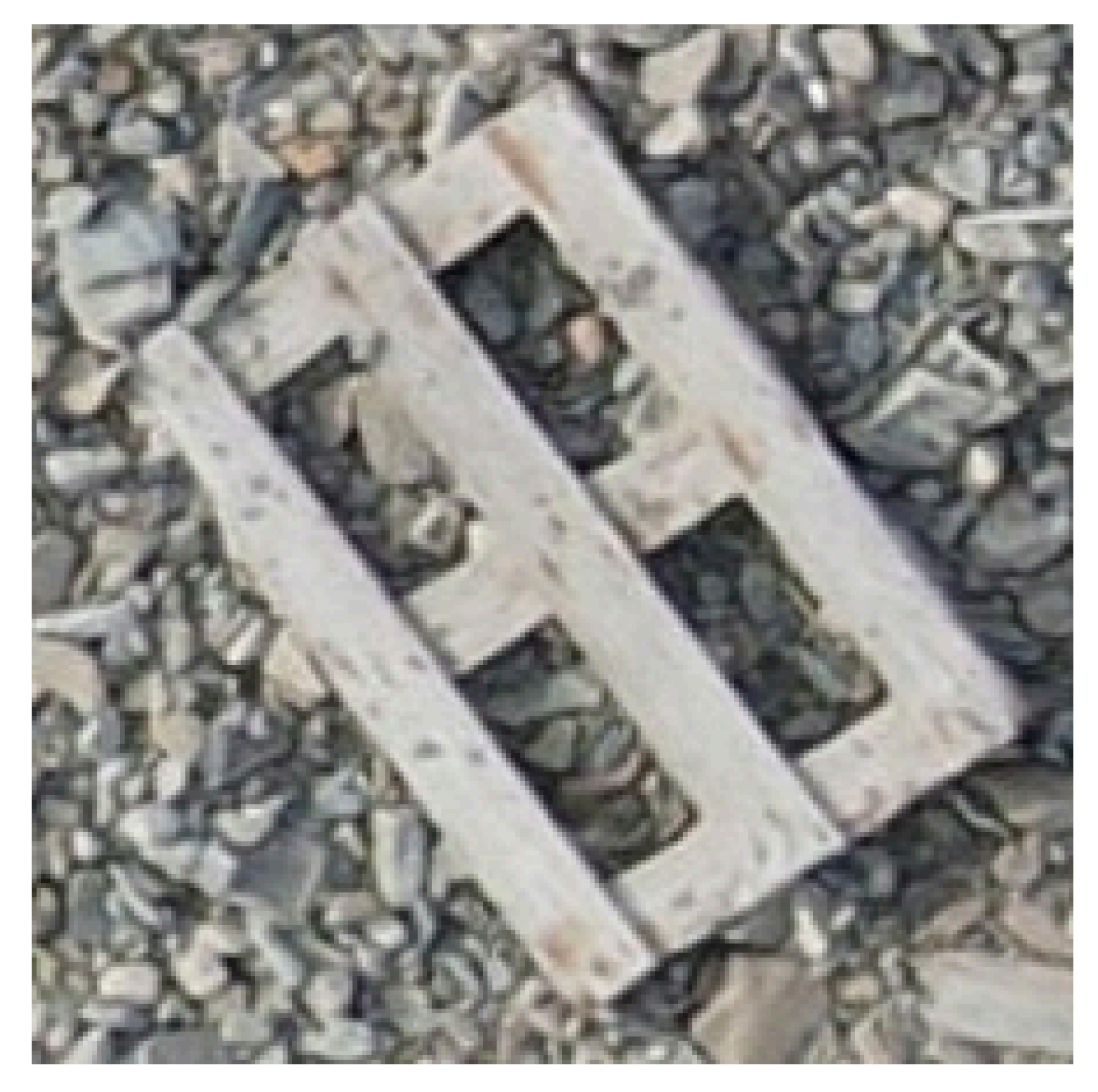
Other	G128, G134, G124	Tires and belts/other rubber pieces/other plastic/polystyrene items (identifiable)	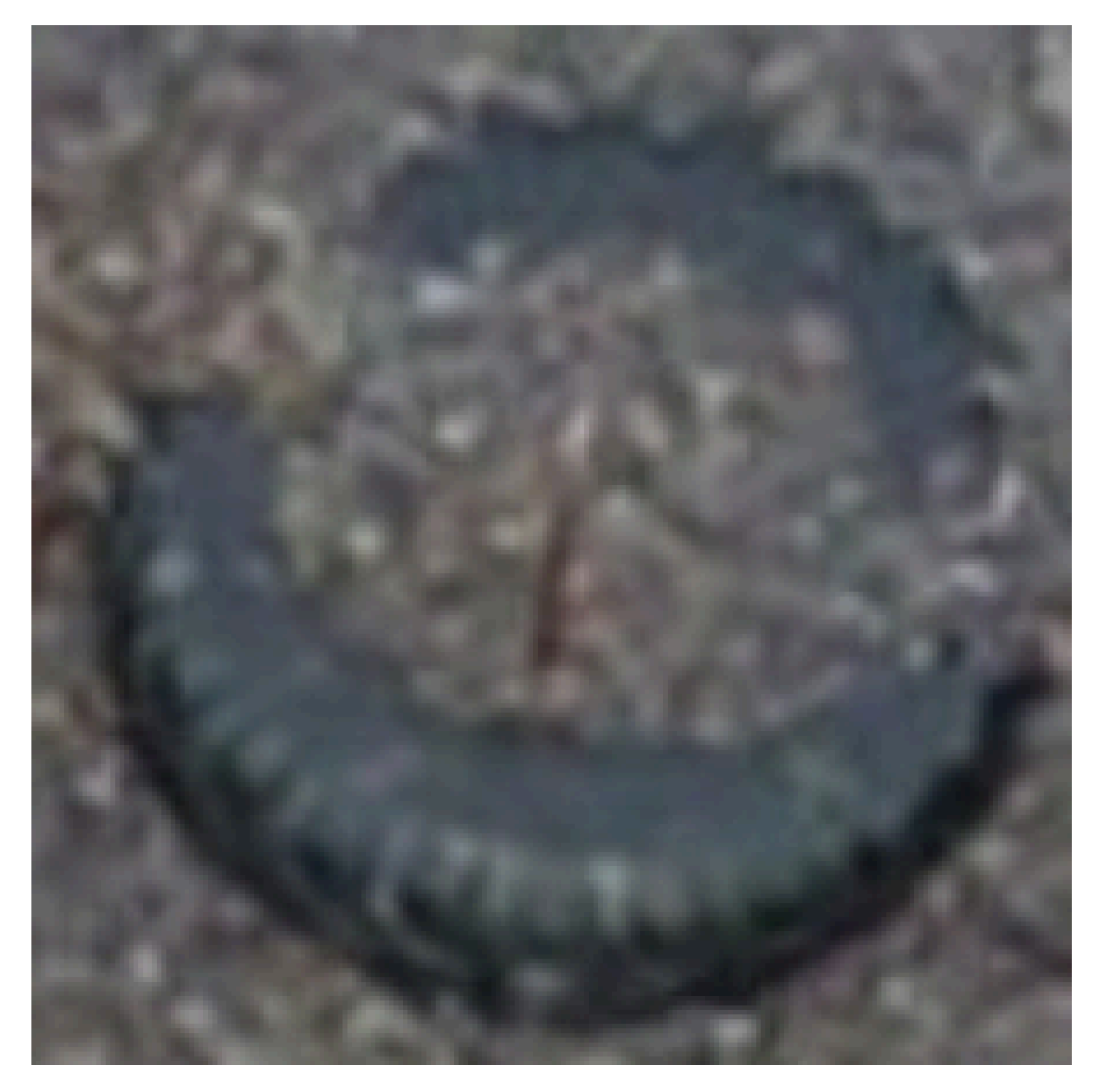

* Note: the code is given according to the MSFD classifier (pp. 164–170 of the Appendix to the Guidance on the Monitoring of Marine Litter in European Seas (2023) [27]).

**Table 3:** Model performance metrics on the validation set (random split).

Class	Class ID	Precision	Recall	mAP50	mAP50-95
Fishing_gear	0	0.813	0.703	0.755	0.477
Metal	1	0.803	0.757	0.811	0.573
Other	2	0.706	0.514	0.556	0.401
Plastic	3	0.820	0.701	0.789	0.548
Wood	4	0.645	0.487	0.551	0.376
Average		0.757	0.632	0.692	0.475

**Table 4:** Model performance metrics on the test set (random split).

Class	Class ID	Precision	Recall	mAP50	mAP50-95
Fishing_gear	0	0.915	0.520	0.630	0.419
Metal	1	0.851	0.627	0.797	0.541
Other	2	0.907	0.522	0.636	0.510
Plastic	3	0.8700	0.632	0.763	0.573
Wood	4	0.857	0.684	0.721	0.513
Average		0.88	0.597	0.709	0.511

**Table 5:** Model performance metrics on the validation set (domain shift split).

Class	Class ID	Precision	Recall	mAP50	mAP50-95
Fishing_gear	0	0.584	0.550	0.523	0.275
Metal	1	0.573	0.489	0.522	0.336
Other	2	0.430	0.360	0.265	0.197
Plastic	3	0.593	0.481	0.514	0.354
Wood	4	0.646	0.339	0.338	0.196
Average		0.565	0.444	0.432	0.272

**Table 6:** Model errors.

Error Type	Number of Errors	Share of Total Errors, %
FN	107	55
FP	72	37
WrongClass	16	8
Total	195	100

**Table 7:** Distribution of errors by class.

Class	Class ID	FN	FP	WrongClass	Total Errors
Fishing_gear	0	18	13	5	36
Metal	1	24	19	2	45
Other	2	31	14	6	51
Plastic	3	15	14	2	31
Wood	4	19	12	1	32
Total		107	72	16	195

## Data Availability

The datasets presented in this article are not readily available because the data are part of an ongoing study. Field data are not publicly available due to restrictions of the Russian law. Requests to access the datasets should be directed to serdukovaelizaveta489@gmail.com.

## Abbreviations

The following abbreviations are used in this manuscript:

UAS	Unmanned aerial system
CNN	Convolutional neural network
AZRF	Arctic zone of the Russian Federation
UNEP	United Nations Environment Programme
ML	Machine learning
AFU	Arctic Floating University
RH	Russkaya Harbour
CZ	Cape Zhelaniya
GSD	Ground Sampling Distance
LH	Ledyanaya Harbour
YOLO	You Only Look Once
OBB	Oriented Bounding Box
mAP	Mean Average Precision
FN	False negative
FP	False positive
